# Analyzing the Spatial Distribution of Immune Cells in Lung Adenocarcinoma

**DOI:** 10.3390/jpm14090925

**Published:** 2024-08-30

**Authors:** Florina Almarii, Maria Sajin, George Simion, Simona O. Dima, Vlad Herlea

**Affiliations:** 1 Department of Pathology, Fundeni Clinical Institute, 022328 Bucharest, Romania; vlad.herlea@umfcd.ro; 2 Department of Pathology, “Carol Davila” University of Medicine and Pharmacy, 050474 Bucharest, Romania; maria.sajin@umfcd.ro; 3 Department of Pathology, Emergency University Hospital, 050098 Bucharest, Romania; george.simion2007@yahoo.com; 4 Department of Surgery, Fundeni Clinical Institute, 022328 Bucharest, Romania; 5 Department of Histopathology, The Center for Excellence in Translational Medicine, 022328 Bucharest, Romania

**Keywords:** NSCLC, tumor microenvironment, immune cell, pathology

## Abstract

(1) Background: This study investigates the tumor immune microenvironment, focusing on immune cell distribution in lung adenocarcinoma. (2) Methods: We evaluated fifty cases of lung adenocarcinoma, and suitable areas for further studies were annotated on the histological slides. Two tumor cores per case were obtained, one from the tumor’s center and another from its periphery, and introduced into three paraffin receptor blocks for optimized processing efficiency. The 4-micrometer-thick tissue microarray sections were stained for H&E and for CD68, CD163, CD8, CD4, and PD-L1; (3) Results: Our investigation revealed significant correlations between PD-L1 expression in tumor cells and the presence of CD163+ macrophages, between CD4+ cells and CD8+, CD68+, and CD163+ cells, and also between CD8+ T cells and CD163+ cells. Additionally, while we observed some differences in cellular components and densities between the tumor center and periphery, these differences were not statistically significant. However, distinct correlations between PD-L1 and immune cells in these regions were identified, suggesting spatial heterogeneity in the immune landscape. (4) Conclusions: These results emphasize the intricate interactions between immune cells and tumor cells in lung adenocarcinoma. Understanding patient spatial immune profile could improve patient selection for immunotherapy, ensuring that those most likely to benefit are identified.

## 1. Introduction

Lung cancer remains the leading cause of cancer-related mortality, with a 5-year relative survival rate of 26.7% reported between 2014 and 2020, according to data from the SEER database [1]. The poor prognosis of this disease is largely attributable to factors such as late-stage diagnosis and compromised patient performance status at presentation [2]. Advanced-stage lung cancer is characterized by significant tumor heterogeneity, both within individual tumors and across different patients, contributing to its complexity and therapeutic resistance [3].

Despite numerous studies assessing tumor heterogeneity in lung adenocarcinoma, we have yet to attain a clear understanding of the changes in the tumor microenvironment (TME) throughout tumor development. Understanding the spatial distribution and densities of immune cells within different compartments of the tumor microenvironment could explain possible interconnections between tumor cells and the immune system. The proximity and arrangement of immune cells relative to tumor cells can significantly influence immune surveillance and the efficacy of immune responses. For example, in one study on lung adenocarcinoma, W. Liu et al. showed that the presence of B cells, a major component of the tumor microenvironment, varied notably during the stages of tumor initiation, invasion, and progression [4]. Various immune responses have been suggested for different stages in tumor progression, as indicated by immunohistochemistry, flow cytometry, and genome and transcriptome analyses [5]. J. Xu et al. showed that the immune profile within the tumor microenvironment changes as lung cancer progresses. Specifically, in adjacent normal tissues, there is an enrichment of naive T cells, while tumor tissues predominantly contain proliferative T cells, cytotoxic T cells, and exhausted T cells. This suggests that T cells in tumor tissues undergo a full immune process from activation to exhaustion. In early-stage patients, there are more naive T cells and fewer exhausted T cells, whereas in late-stage patients, there is an increased presence of proliferative, cytotoxic, and exhausted T cells, indicating more active T cell-mediated immune responses as the tumor advances [6].

This is an important reason why immunotherapy, particularly anti-PD1-PD-L1 drugs, which depend on T cell-mediated adaptive immunity, is expected to work better in patients in advanced stages [7].

Significant advancements in molecular analyses have deepened our comprehension of cancer cell evolution towards immune evasion [8]. Characterization of immune checkpoint pathways, such as PD-1/PD-L1 and CTLA-4, has been pivotal in this regard, elucidating how cancer cells suppress immune responses. These insights have been instrumental in the development of immunotherapies, which aim to restore and enhance the immune system’s ability to recognize and eliminate tumor cells [9].

Immunotherapy, particularly immune checkpoint inhibitors (ICIs), has revolutionized the treatment landscape of lung adenocarcinoma, offering hope for durable responses and improved survival rates. However, the variability of responses to immunotherapy highlights significant gaps in our understanding of the tumor microenvironment (TME) and the spatial arrangements of immune cells within it [10]. These spatial and density variations in immune cells can provide valuable insights into the tumor–immune dynamics and potentially inform therapeutic strategies, improving prognostic outcomes for lung adenocarcinoma patients.

Intratumor heterogeneity is an important aspect of cancer biology that influences tumor behavior, treatment response and patient outcomes. H.J. Wu at. al. demonstrated that both the cellular composition of the tumor microenvironment and the genomic heterogeneity of individual tumor cells contribute to the spatial diversification of human lung adenocarcinoma. Importantly, this spatial diversification was found to significantly correlate with patient survival, highlighting the clinical relevance of understanding intratumor heterogeneity in the context of tumor biology [11].

Knowledge of spatial heterogeneity can lead to the development of delivery systems that target specific tumor regions, maximizing therapeutic efficacy while minimizing off-target effects. Also, studying the spatial distribution of immune-suppressive cells (e.g., M2 macrophages) can reveal mechanisms of immune evasion and resistance, guiding the development of combination therapies to overcome these barriers.

Current research endeavors are increasingly focused on unraveling the intricate dynamics within the tumor microenvironment, recognizing its pivotal role in both tumor progression and regression [12].

Given these considerations, the aim of the present study is to investigate the spatial distribution of immune cells in lung adenocarcinoma by comparing the tumor center and margin. By providing a detailed analysis of immune cell localization and density, the research seeks to address critical gaps in our understanding of the interactions between tumor and immune cells. This study seeks to enhance our understanding of TME compartmentalization and contribute to the development of more effective and personalized treatment strategies for lung adenocarcinoma.

## 2. Materials and Methods

### 2.1. Study Design

**Figure 1:**
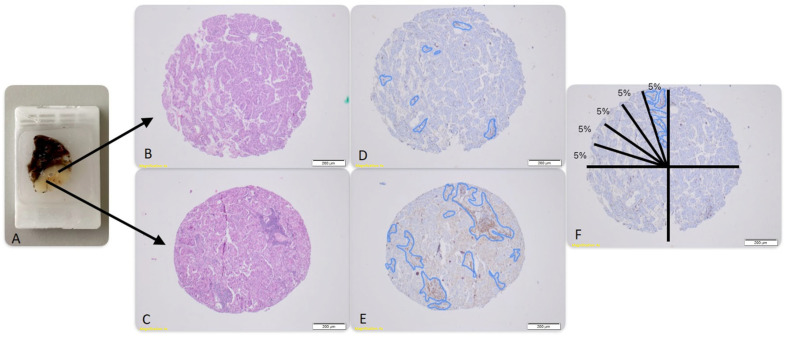
(**A**) Shows a paraffin block from a patient with lung adenocarcinoma. Two cores were obtained from the spots marked by the base of the arrows, and the H&E-stained cores are shown in pictures (**B**) (middle of the tumor) and (**C**) (margin of the tumor). CD8 staining is illustrated in pictures (**D**) and (**E**), showing 5% positivity in the middle of the tumor and 40% positivity at the periphery. Picture (**F**) shows the algorithm used for appreciating infiltrating cell density as a percentage of the core surface.

In this study, we included fifty cases of lung adenocarcinoma treated at the University Emergency Hospital of Bucharest during the period of October 2016–August 2020. The cases were selected after examining the histological slides, and the ones that included at least one slide featuring tumor and adjacent non-tumoral lung tissue were chosen. This approach ensured the inclusion of both tumor and normal tissue regions for comprehensive analysis. We gathered the pathological result, all the available clinical data and one paraffin block for every patient and we undertook further tests at Fundeni Clinical Institute and The Center of Excellence in Translational Medicine, Bucharest. Representative sampling was conducted by reviewing the histological slides, annotating the tumor border, and identifying the furthest point towards the tumor’s center with optimal malignant cell density. This method facilitated the inclusion of both the tumor margin and central regions while avoiding areas of pure necrosis. Two tumor cores per case were collected from selected regions—one from the tumor’s center and another from its periphery (Figure 1). All cores were embedded into three paraffin receptor blocks using Alphelys’ Tissue Arrayer MiniCore^®^ 3 hardware solution to optimize processing efficiency and examination time. The resulting 4 micrometer thick tissue microarray sections, with 4 μm in thickness, were stained for H&E and with Vitro Master Diagnostica^®^ markers using the fully automated MD Stainer Platform (Vitro, S.A, Sevilla, Spain). The antibodies used were CD68 (clone Kp-1, dilution 1:50, incubation, 25 °C for 10 min), CD163 (clone EP324, dilution 1:50, incubation, 25 °C for 20 min), CD4 (clone EP204, dilution 1:50, incubation, 25 °C for 30 min), CD8 (clone SP16, dilution 1:50, incubation, 25 °C for 30 min), and PD-L1 (clone Cal10, dilution 1:50, incubation, 25 °C for 60 min). For each receptor block, one core of tonsil tissue was included as a positive control in order to ensure staining specificity.

### 2.2. Marker Scoring Protocols

To establish the spatial profile of the immune process within the tumor, the slides were evaluated by two pathologists. Cell quantification was performed manually, adapting the VENTANA Interpretation Guide for the PD-L1 (SP142) Assay to CD68, CD163, CD4, and CD8 markers. As shown in Figure 1, the areas occupied by positive immune cells were reported as a percentage of the total surface area of the core. PD-L1 slides were scored based on the Interpretation Guide for PD-L1 (SP263) Assay, accessible online, which evaluates the percentage of tumor cells (%TC) with any membrane expression, also known as the TPS score.

### 2.3. Statistical Analysis

All statistical analyses were performed using GraphPad Prism V.10.2.0 software (GraphPad Software, Boston, MA, USA, www.graphpad.com, accessed on 1 June 2024). Cell percentage scores, patient age, and tumor diameter were correlated using Spearman’s rank correlation test. For statistical comparison of marker expression between the middle and the margin of the tumor, the Wilcoxon signed-rank test was used. Comparison between multiple non-parametrical groups was conducted by applying Kruskal–Wallis’s test in order to assess whether there were differences in immune cell counts. A *p* value of less than 0.05 was considered statistically significant.

## 3. Results

The research cohort encompassed a total of 50 individuals, with the specific attributes of each patient detailed in Table 1. Following the verification of congruence in histological subtypes between core biopsy samples and initial diagnostic assessments via examination of hematoxylin and eosin (H&E)-stained slides, immunohistochemical analyses were conducted. Immunohistochemistry assays facilitated the assessment of immune cell markers utilizing the scoring methodology delineated in Figure 1.

We evaluated the cellular component densities in both the tumor center and the periphery. Despite observing some visual differences in cell densities between these regions, statistical analysis using the Wilcoxon signed-rank test indicated no significant difference in the mean positive cell densities between the tumor center and margin.

**Figure 2:**
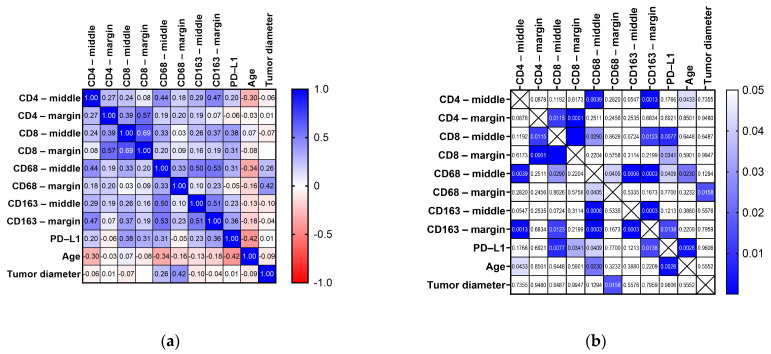
Analysis of the cell positivity for CD4, CD8, CD68, and CD163 in the middle and at the margin of the tumor, as well as with PD-L1 expression, patient age, and tumor diameter illustrated as: (**a**) Spearman R correlation matrix; (**b**) *p* value correlation matrix.

However, multiple correlations were identified between immune marker expressions and their localization, as well as with PD-L1, patient age, and tumor diameter. Figure 2 illustrates these correlations along with their respective *p*-values. Statistically significant correlations, indicated by *p*-values < 0.05, are highlighted in Figure 2b.

We observed that the cell densities for CD8, CD68, and CD163 were significantly correlated between the center and the margin of the tumor (*p* values < 0.0001, 0.0405, and 0.0003), while CD4 did not show any such correlation (*p* value = 0.0878). 

Within the tumor center, CD68 counts were correlated with CD4, CD8, and CD163 of the same region. This indicates a possible interaction between macrophages and both T cell subsets (CD4+ and CD8+), as well as other macrophages (CD163+), suggesting that macrophages in the tumor center might play a central role in coordinating the immune response.

Additionally, CD68 in the middle of the tumor exhibited moderately positive and significant correlations with CD163 at the margin (*p* value = 0.0003) and PD-L1 expression (*p* value = 0.0409).

Interestingly, CD163 at the tumor border correlated with all the markers from the middle core and also with PD-L1 expression, whereas CD163 in the center correlated only with CD163 at the periphery. This highlights the potential role of peripheral M2 macrophages in modulating immune responses across the tumor.

CD4 at the margin, as well as PD-L1, correlated with CD8 expression in both regions (margin and center).

Age showed a moderately negative and significant correlation with CD4 and CD68 in the middle, as well as with PD-L1 expression. This implies that older patients may have a decreased presence of CD4+ T cells and macrophages in the tumor center, along with lower PD-L1 expression, which could impact the efficacy of immune responses and immunotherapy.

Tumor diameter was only correlated with the expression of CD68 at the margin of the tumor (*p* value = 0.0158), and no additional clinicopathological differences were identified when comparing the maximum expression of immune markers between males and females, left-sided and right-sided tumors, or acinar and non-acinar lung adenocarcinomas.

## 4. Discussion

The tumor microenvironment plays a pivotal role in the development and progression of carcinomas, influencing their behavior and response to treatment. In 2009, Camus et al. [13] described three immune profiles of colorectal carcinomas, calling them “hot, variable and cold”. The “hotness” or “coldness” of tumors is determined by various factors, including the characteristics of cancer cells themselves, the tumor immune profile, the tumor microenvironment, and signaling mechanisms. These factors play crucial roles in determining the clinical efficacy of cancer treatments for patients [12].

Tumors with a tumor microenvironment rich in infiltrating lymphocytes, overexpression of PD-L1, genomic instability, and high tumor mutation burden are considered “hot tumors” [14]. Tumors with high tumor mutational burden typically harbor increased levels of neoantigens, which can be targeted and recognized by the immune system. This is why such tumors often exhibit more favorable responses to immune therapy strategies [15].

The Cancer Genome Atlas (TCGA) projects on the lung showed that the mutational burden of lung adenocarcinomas developed in smokers occurs throughout the whole genome and is significantly higher than in former or never-smokers [16]. Genomic studies comparing the TME in smokers and non-smokers showed that immune-related genes and markers for activated CD4- and CD8-positive cells, have significantly higher expression in smokers. By means of mass cytometry, Sun Y. et. al. showed that the smoking group had an activated immune microenvironment, and this could be the reason for the longer progression-free survival period under ICI for smokers. This is related to the high mutation burden and the subsequent increased load of neoantigens [17]. One of the genes alterations frequently associated with smoking is TP53. TP53, the gene encoding the protein p53, an important tumor-suppressive transcription factor, is one of the most prevalent mutations in lung cancer, with up to 46% of lung adenocarcinoma being included [18]. The tumors harboring this mutation exhibit a particular tumor microenvironment characterized by elevated PD-L1 expression and an increased presence of CD8+ T cells, indicating a highly immunogenic state and adaptive immune resistance [19].

In their study, Chen et al. provided a comprehensive analysis of the immune landscape in lung adenocarcinoma across different disease stages. Using single-cell RNA sequencing and bulk RNA sequencing analyses, they demonstrated significant variations in CD8+ cytotoxic T lymphocytes, regulatory T cells, and follicular B cells between early and advanced stages of the disease. The authors also highlighted intratumor heterogeneity, showing that cells from advanced-stage lung adenocarcinoma exhibited higher copy number variations (CNVs) compared to early-stage tumor cells or normal tissue [20].

In our study, we demonstrated a statistically significant correlation between PD-L1 expression of the tumoral cells and CD163-positive macrophages, supporting the complex protumoral interactions in lung adenocarcinomas, by harnessing the immune suppression potential of M2 macrophages (CD163+) and tumor immune evasion mechanisms. An immune cell exhaustion could explain the positive correlation between CD8+ and CD163+ cells. These associations were described in other studies on non-small cell lung carcinoma [21,22], as well as in other tumor types, such as oral in situ and invasive carcinoma [23], cervical carcinoma [24], or melanoma [25]. Shima et al. proposed that tumor-associated macrophages act as external regulators of PD-L1 expression in lung adenocarcinoma. This finding suggests that a combined therapeutic strategy targeting both tumor PD-L1 and tumor-associated macrophages may be an effective approach for treating lung cancer [22]. T cells, especially CD8+ T cells, become dysfunctional after long periods of interactions with tumor cells, and this state is defined as T cell exhaustion [26]. These cells present overexpressed inhibitory receptors, loss of effector function, and even the capacity to induce immune tolerance [27], and they are associated with an increased number of CD163+ cells in other tumor types as well [28].

A high level of lymphocytes abundance was associated with favorable overall survival in lung adenocarcinoma, and a significantly better prognosis was observed in patients with preferential homing of lymphocytes to the periphery of the tumor [29]. A comprehensive analysis of the tumor immune microenvironment in the tumor center and invasive margin of lung non-small cell carcinoma revealed that T cell density is higher at the invasive front, where the proximity to tumor cells is greatest [30].

Studies have shown that densities, as well as distribution patterns and proximity of the inflammatory cells with the tumoral cells, could be associated with different outcomes. For instance, a close relation of CD8+ activated cytotoxic T cells and malignant squamous cell in NSCLC was associated with better relapse-free survival, playing a role in preventing tumor recurrence [31]. The prognosis of patients with lung carcinoma was found to be correlated with various cell phenotypes. Multiple cells have shown immunosuppressive activity in lung carcinoma, such as antigen-experienced T-cells positive for PD-L1, regulatory T-cells, and T-cells expressing B7-H3 [32,33,34,35]. Macrophages expressing B7-H4 showed positive correlations with the recurrence rate of patients with NSCLC [15], recognizing their roles in tumor genesis, tumor development, and metastasis [36]. The spatial analysis and mapping of the cell phenotypes allows for the distinction of various cell populations that exhibit specific roles in activation and regulation of the intratumoral immune pathways. Thus, the distribution of these cells suggests that they may fulfill specific roles based on their spatial organization [37]. The heterogeneity of the tumor microenvironment may contribute to the limited efficacy of monotherapy targeting single inhibitory pathways, potentially explaining why only certain patients respond to such treatments [38].

Through this study, we proposed a surrogate approach for computational pathology to examine immune cell heterogeneity and spatial distribution across tumors in lung adenocarcinoma. Our study revealed notable correlations between and variations in the markers under investigation. PD-L1 expression correlated with the presence of CD8+T cells irrespective of their location, showing a stronger correlation in the tumor center, with CD68+ macrophages only in the tumor center and with CD163+ macrophages only at the tumor margin. This observation underscores the immune cell heterogeneity in the tumor microenvironment.

Previous studies have extensively analyzed the interactions between immune cells and PD-L1 in various cancers, including lung adenocarcinoma. One in vitro study demonstrated the inhibitory effect of soluble PD-L1 variants on CD4+ and CD8+ T cell activation, showing reduced activation in the presence of PD-L1 fusion proteins compared to controls [39].

Spatial transcriptomics in lung carcinoma further revealed the close proximity of macrophages, CD8+ T cells, and PD-L1, suggesting that these spatial patterns contribute to T cell exhaustion [40].

Noteworthy correlations were also detected between other immune markers, such as CD4 and CD8, indicating interplay between different immune cell populations. It has been reported that IL-21 produced by CD4+ T helper cells enhances the effector functions of CD8+ tumor-infiltrating lymphocytes (TILs) through IL-21/IL-21R signaling [41,42]. Using a non-T cell receptor transgenic, immunocompetent tumor model, researchers demonstrated that the eradication of aggressive cancers through T cell targeting of tumor stroma crucially depends on the cooperation between CD4+ and CD8+ T cells. In this study, the cooperation was essential during both the induction and effector phases in the tumor microenvironment [43]. In our study, the correlation between CD4+ T cells at the periphery and CD8+ T cells throughout the tumor, coupled with the lack of correlation of central CD4+ T cells, highlights the complex and spatially variable nature of the tumor immune microenvironment in lung adenocarcinoma.

We found a moderately positive and statistically significant correlation regarding tumor dimension and CD68 expression at the tumor border. Studies have shown that high expression levels of CD68 are significantly and positively related to the number of neoantigens in lung adenocarcinoma [22]. Neoantigens are foreign proteins that are absent in normal tissues and can emerge in tumors through various mechanisms, including genomic mutations, aberrant transcriptomic variants, or post-translational modifications [44]. Recent advances in neoantigen research have significantly accelerated the development and regulatory approval of tumor immunotherapies, such as cancer vaccines [45], adoptive cell therapy, and antibody-based therapies [44,46].

The lack of significant differences in cell density between the center and the margin of the tumor for CD8-, CD68-, and CD163-positive cells suggests a relatively uniform distribution of immune cells within the selected cores. Several studies evaluating various tumor types have also reported no differences in immune cell distribution between the tumor center, tumor stroma, and transition zone [47,48]. This uniformity might indicate that immune responses could be similarly effective throughout the tumor; these cells might have more uniform migration and infiltration patterns across the tumor, or they may be driven by similar chemotactic signals throughout the tumor tissue. In contrast, CD4+ T cells could exhibit more localized migration and activation patterns. These cells may be recruited to specific regions where their functions are needed, resulting in less uniform distribution. However, the absence of significant spatial heterogeneity in immune cell densities may be related to the sampling methodology, where the distance between cores taken from the same tissue section could be too small to accurately assess the differences between the central and peripheral regions of the tumor.

Furthermore, the oxygen gradient within the tumor microenvironment (TME) profoundly influences the biological behavior of various immune cells, affecting their infiltration, migration, polarization, function, and metabolism [49]. M1 macrophages, known for their antitumor properties, are typically found in normoxic areas near blood vessels, usually at the periphery, whereas M2 macrophages, which promote tumor growth, are more prevalent in the hypoxic tumor center [50,51]. The functions of other immune cells are also influenced by the oxygen gradient. For instance, neutrophils, natural killer (NK) cells, and regulatory T cells (Tregs) migrate toward hypoxic regions, while CD8+ T cells tend to remain in oxygen-rich areas [52]. Lung adenocarcinoma is characterized by its morphological diversity, and a corresponding spatial heterogeneity of the microenvironment is anticipated. Variations within a single tumor can be extensive, with different regions exhibiting varying levels of immune cell infiltration, necrosis, and vascularization [53]. While two cores might capture some of this variation, significant areas of heterogeneity may remain unsampled.

To validate the findings, future studies should include larger cohorts. This would help to determine whether the observed trends hold true across a more diverse patient population and reduce the impact of individual tumor variability. Increasing the number of sampled cores per tumor could improve the representation of spatial heterogeneity. Sampling from multiple distinct regions and different paraffin blocks would provide a more accurate picture of the tumor microenvironment. While two cores from each tumor provide some insights into the spatial variability within lung adenocarcinoma, this approach has limitations in fully capturing the complexity of the entire tumor. Utilizing whole-slide imaging and analysis can provide a more comprehensive view of the tumor landscape. Techniques such as multiplex immunohistochemistry or immunofluorescence and digital pathology allow for the assessment of immune cell densities across entire tissue sections, minimizing the limitations associated with core sampling.

## 5. Conclusions

In summary, our study provides valuable insights into the immune microenvironment of lung adenocarcinoma by analyzing immune cell densities in the tumor center and margin. The findings highlight critical aspects of spatial immune cell distribution that could influence therapeutic responses. However, several limitations of our study should be acknowledged.

While our correlations are supported by statistical analyses and are consistent with existing literature, the lack of experimental validation remains a significant limitation. Additionally, the study focused on comparing immune cell densities between the tumor center and periphery, but did not include a detailed analysis of cell patterning. Addressing this limitation could provide deeper insights into the spatial organization and interactions of immune cells within the tumor microenvironment.

Future research should address these limitations by employing larger cohorts; comprehensive clinicopathological profiles; and advanced techniques like multiplex immunohistochemistry, immunofluorescence, and single-cell RNA sequencing. Incorporating detailed spatial analyses will offer a more robust understanding of tumor heterogeneity and its impact on immunotherapy response, potentially leading to more effective and personalized lung cancer treatments.

## Figures and Tables

**Table 1:** Characteristics of the patients included in this study and correlation of biomarker expression with clinicopathological attributes.

Characteristics	Categories	Total *n* (%)		Biomarker Correlation (*p*-Value)							
			CD68		CD163		CD4		CD8		PD-L1
Age (*n* = 50)	<50	7 (14%)	Center	Margin	Center	Margin	Center	Margin	Center	Margin	
	51–60	14 (28%)	*p =* 0.0230	*p* = 0.3232	*p* = 0.3880	*p* = 0.2209	*p* = 0.0433	*p* = 0.8501	*p* = 0.6448	*p* = 0.5901	*p =* 0.0026
	61–70	19(38%)									
	>70	10 (20%)									
Sex (*n* = 50)	female	11 (22%)	*p* = 0.7591		*p* = 0.5075		*p* = 0.2849		*p* = 0.8532		*p* = 0.5308
	male	39 (78%)									
Tumor laterality	left	17 (34%)	*p* = 0.8838		*p* = 0.8091		*p* = 0.9837		*p* = 0.4385		*p* = 0.5127
	right	33 (66%)									
Tumor dimension	<3 cm	26 (52%)	*p* = 0.1294	*p =* 0.0158	*p* = 0.5576	*p* = 0.7959	*p* = 0.7355	*p* = 0.9480	*p* = 0.6487	*p* = 0.9947	*p* = 0.9606
	3–5 cm	9 (18%)									
	5–7 cm	11 (22%)									
	>7 cm	4 (8%)									
Histological subtype	Acinar	34 (68%)	*p* = 0.7494		*p* = 0.3760		*p* = 0.9455		*p* = 0.7784		*p* = 0.1591
	Solid	7 (14%)									
	Mucinous	4 (8%)									
	Adenosquamous	2 (4%)									
	Pleomorphic	2 (4%)									
	Intestinal	1 (2%)									

## Data Availability

The datasets used and/or analyzed during the current study are available from the corresponding author upon reasonable request.
